# Laser hair depilation for the prevention of disease recurrence in adolescents and young adults with pilonidal disease: study protocol for a randomized controlled trial

**DOI:** 10.1186/s13063-018-2987-7

**Published:** 2018-11-01

**Authors:** Peter C. Minneci, Devin R. Halleran, Amy E. Lawrence, Beth A. Fischer, Jennifer N. Cooper, Katherine J. Deans

**Affiliations:** 1 Center for Surgical Outcomes Research, The Research Institute at Nationwide Children’s Hospital, 700 Children’s Drive, FB 3A.3, Columbus, OH 43205 USA; 2 Department of Pediatric Surgery, Nationwide Children’s Hospital, Columbus, OH USA

**Keywords:** Pilonidal disease, Laser hair depilation, Recurrence, Randomized controlled trial

## Abstract

**Background:**

Laser hair depilation is a promising therapy in the management of pilonidal disease. However, the large controlled trials needed to demonstrate the effectiveness of this practice have not been performed.

**Methods:**

We designed a single-center randomized controlled trial that will enroll 272 patients with pilonidal disease. Patients will be randomized to receive laser hair depilation of the sacrococcygeal region or the best recommended standard of care. The primary outcome is the rate of recurrent pilonidal disease at 1 year, defined as development of a new pilonidal abscess, folliculitis, or draining sinus after treatment, which would require antibiotic treatment, additional surgical incision and drainage, or excision within 1 year of enrollment. Secondary outcomes include each of the following at 1 year: disability days of the patient, disability days of the caregiver, health-related quality of life, healthcare satisfaction, disease-related attitudes and perceived stigma, pilonidal disease-related complications, pilonidal disease-related procedures, surgical excision, postoperative complications, and compliance with recommended treatment.

**Discussion:**

This study will determine the effectiveness of laser hair depilation to reduce pilonidal disease recurrence in adolescents and young adults as compared to the best recommended standard of care.

**Trial registration:**

ClinicalTrials.gov, NCT03276065. Registered on 8 September 2017.

**Electronic supplementary material:**

The online version of this article (10.1186/s13063-018-2987-7) contains supplementary material, which is available to authorized users.

## Background

Pilonidal disease is a common infectious condition in the adolescent and young adult population, affecting approximately 1% of individuals aged 15–30 years [1–3]. The disease is believed to be caused by the insertion of loose hairs into the natal cleft, resulting in a chronic foreign body reaction and the formation of epithelialized tracts and midline pits [4]. Guidelines for the medical management of pilonidal disease support meticulous hygiene to the sacrococcygeal area and routine hair removal by mechanical or chemical depilation [5]. However, compliance with these recommendations is low, and patients frequently suffer considerable morbidity related to disease recurrence [6].

Laser hair depilation has been studied as a strategy to decrease pilonidal disease recurrence rates. Several studies have demonstrated the efficacy of laser hair depilation to reduce pilonidal disease recurrence compared to standard of care in both adults and children [7–18]. These studies conclude that laser hair depilation is a useful adjunct in preventing recurrence; however, most of these studies were small, retrospective, or not sufficiently controlled, and many of the authors have called for additional well-controlled prospective randomized controlled trials (RCTs) in a broader population [11, 15, 19, 20].

We therefore designed a prospective, RCT to determine the effectiveness of laser depilation in reducing pilonidal disease recurrence compared to the best recommended standard of care. In addition to recurrent disease, this trial will evaluate other outcomes important to patients, including disease-related disability, the need for procedures such as surgical excisions or drainage procedures, health-related quality of life (HRQOL), healthcare satisfaction, and disease-related attitudes and perceived stigma.

## Methods

### Study design

This is a prospective RCT comparing two currently available treatment options for patients with pilonidal disease. The control group receives the best recommended standard of care. The intervention group receives laser therapy in addition to the best recommended standard of care. The control group treatment regimen is based on recommendations from published studies and guidelines [3, 5, 15, 21, 22]. The intervention group treatment regimen was designed based on the interventions described in previously published studies and a pilot study we previously performed at Nationwide Children’s Hospital (NCH) [7–17, 23].

### Stakeholder team

As this study was designed to assess outcomes related to pilonidal disease that are important to patients and their families, we designed this trial with significant input from a multi-disciplinary stakeholder group that includes patients, caregivers, community-based pediatricians, emergency medicine physicians, adult and pediatric surgeons, and nurses. We engage our stakeholder partners through in-person individual interviews and stakeholder group meetings to discuss planning and conducting the study and disseminating results. In-person stakeholder meetings are held semiannually to provide insight into issues related to study design, recruitment, retention, study progress, and developing plans for dissemination of the results.

### Study population

Inclusion and exclusion criteria for the trial are listed in Table 1. Patients aged 12–21 years with a diagnosis of pilonidal disease are eligible for participation. The inclusion criteria were chosen to capture patients with the highest incidence of pilonidal disease and who have the highest likelihood of tolerating the laser treatment. In addition, all patients with pilonidal disease are included without regard for the number of previous episodes of disease or surgical history. Patients with a history of photosensitivity are ineligible for the study, and those with an acutely inflamed pilonidal sinus, cyst, or abscess are invited to participate upon resolution of their acute episode of disease. After determining that all eligibility criteria are met, a trained member of the research team invites the patient and legal guardian to enroll.

**Table 1:** Eligibility criteria

Inclusion criteria	
• Age 12–20 years	
• Diagnosis of pilonidal disease	
Exclusion criteria	
• History of photosensitivity	
• Actively inflamed pilonidal sinus (these patients will be informed of the trial and invited to contact the study team upon resolution of their inflamed sinus if they are interested in being in the trial at that time)	

### Study setting

This trial is underway at our institution. Adolescents and young adults with pilonidal disease in the Columbus, Ohio metropolitan area are recruited from the clinics, emergency departments, and inpatient units at NCH and the Ohio State University (OSU) Wexner Medical Center, and from the clinics of community pediatric practices.

### Randomization methods

Our randomization sequence was created using a randomized block scheme, with blocks of size four or six selected randomly with equal probability. The sequence was built using the plan procedure in SAS 9.4 (SAS Institute Inc., Cary, NC, USA). It was generated and is maintained by the project statistician and is unavailable to other research study members. After study staff verify patient eligibility (further described below) and obtain informed consent, patients wishing to enroll in the trial are randomized within the web-based Research Electronic Data Capture (REDCap) system using the earlier generated randomization list [24].

### Baseline assessment

After verification of eligibility by initial phone interview and again at the first clinic visit prior to randomization and treatment, both the patient and legal guardian are asked to provide demographic and socioeconomic information including age, race, ethnicity, gender, annual household income range, patient occupation (from those engaged in paid employment), guardian occupations, and specific insurance coverage. We also collect a complete medical history of pilonidal disease, family history of pilonidal disease, current pilonidal disease symptoms, current hygiene regime, and history of physician office visits, emergency department/urgent care visits, and inpatient hospitalizations for pilonidal disease. A sample list of the collected data points is provided in Table 2. At the initial clinic visit, a physician member of the research team (1) reviews the study protocol and procedures, (2) explains the risks and benefits of each treatment, (3) answers questions, and (4) obtains written informed consent and assent (for patients < 18 years of age). The child and legal guardian are asked to complete the age-appropriate Pediatric Quality of Life Inventory (PedsQL™) and questions about healthcare-associated disability, pain, and pilonidal disease management. The patient also completes the Child Attitude Toward Illness Scale (CATIS), and the parent and child complete the disease-related stigma scales. Patients are then randomized and informed of their treatment arm.

**Table 2:** Sample of data to be collected

• Demographics (including age, gender, body mass index, race, ethnicity, caregiver education level, employment status, income, marital status, number of household residents)	
• Fitzpatrick skin type	
• Vital signs	
• Laboratory testing	
• Episodes of pilonidal disease	
• Surgical procedures performed for pilonidal disease	
• Symptoms	
• Medications	
• Complications (if any)	
• Readmissions	
• Recurrence	
• Days missed from work or school	
• Days missed from normal activities	
• Office visits referable to pilonidal disease	
• Emergency department visits for pilonidal disease	
• Treatment-related pain	

### Treatment arms

#### Control group

Subjects randomized to the control group receive standardized education and training about hair removal, reflective of the best recommended standard of care. Patients and families in the control group are taught hair removal techniques, and the technique of mechanical depilation is demonstrated for the patient and caregiver with the nurse or physician shaving the gluteal cleft of the patient during this visit. It is recommended that they perform either chemical or mechanical depilation as needed to keep the area hair-free. They are given supplies in clinic to be able to perform hair removal for the next 6 months. Furthermore, the patient and family are given the option to schedule additional in-person visits for further education and training on hair removal as desired.

#### Intervention group

Similar to the control group, the patients and families in the intervention group are taught hair removal techniques at the initial visit and asked to perform either chemical or mechanical depilation as needed to keep the area hair-free between clinic treatments. Fitzpatrick skin type classification is assessed at the first treatment visit and used to select the best laser to perform hair removal for each patient [7–17, 23]. A 7% lidocaine/7% tetracaine cream is applied 45 min prior to treatment to minimize any discomfort associated with the heat of the laser treatments. Patients receive either an 810 nm (for Fitzpatrick skin types I–IV) or Nd:YAG (for Fitzpatrick skin types V–VI) 28 joule application at auto pulse duration for 400 ms. Subjects randomized to the laser depilation arm visit the surgery clinic for one treatment every 4–6 weeks to receive a total of five treatments. The energy settings of the laser are sequentially increased at each visit in order to maximize the depilation effect.

### Outcomes

The primary outcome is pilonidal disease recurrence at 1 year, defined as development of a new pilonidal abscess, folliculitis, or draining sinus after treatment, which would require antibiotic treatment, additional surgical incision and drainage, or excision. Secondary outcomes include each of the following at 1 year: disability days of the patient, disability days of the caregiver, HRQOL, healthcare satisfaction, disease-related attitudes and perceived stigma, pilonidal disease-related complications, pilonidal disease-related procedures, surgical excision, postoperative complications, and compliance with recommended treatment.

### Assessments and follow-ups

**Fig. 1:**
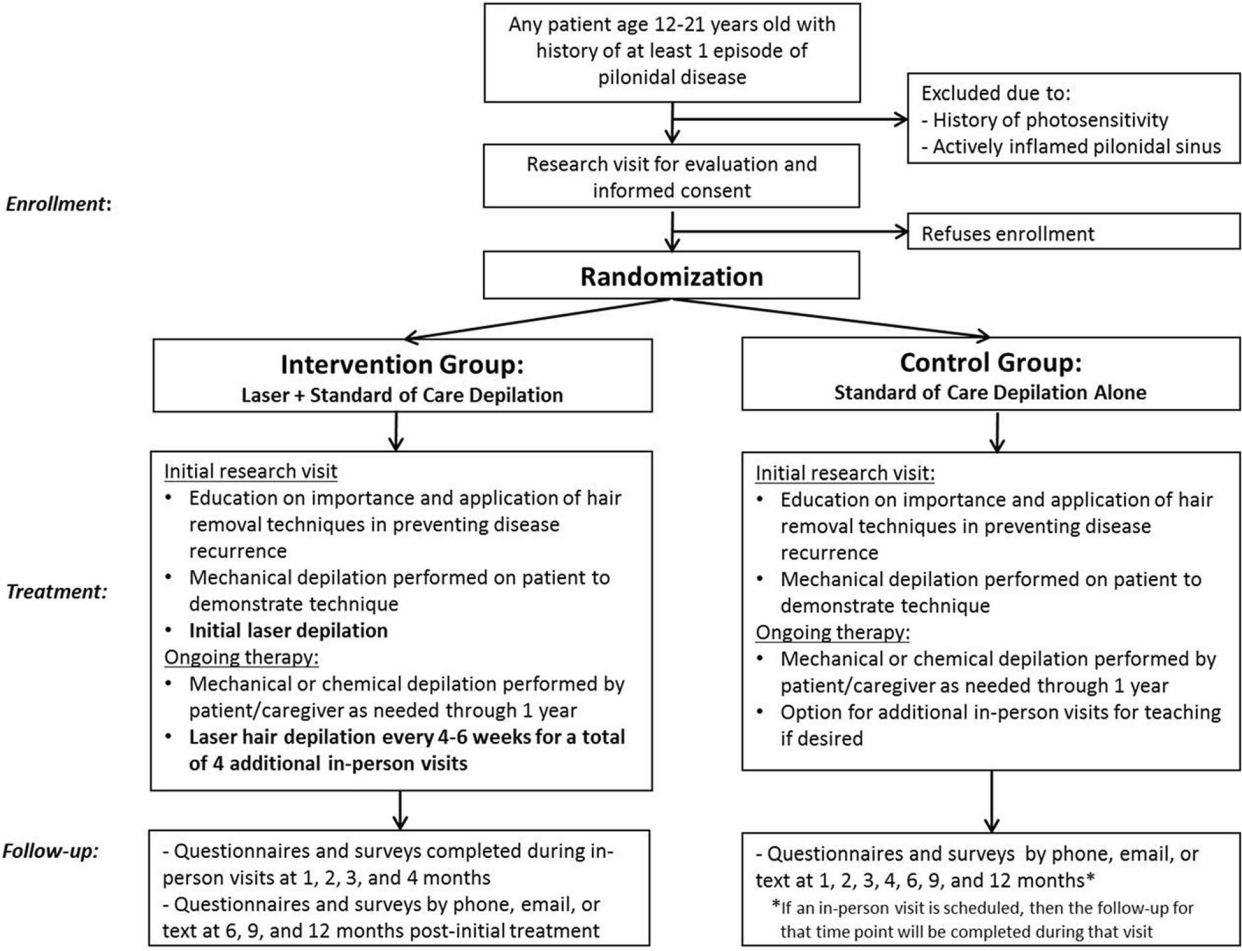
Study overview

**Fig. 2:**
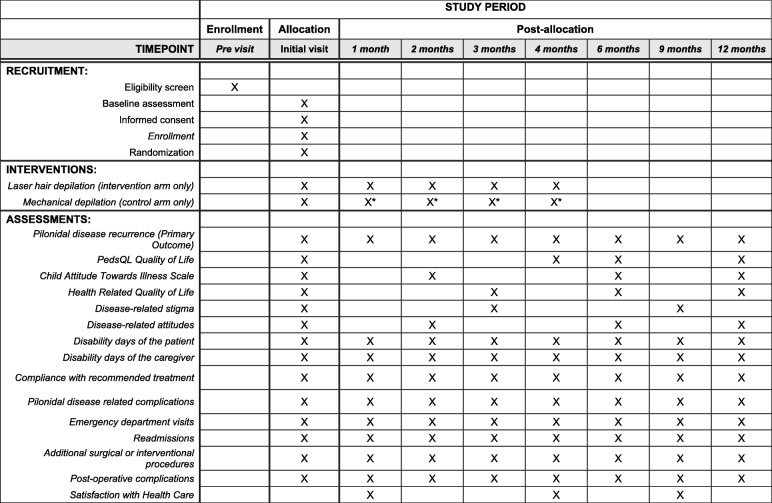
Schedule of enrollment, interventions, and assessments over the study period (SPIRIT statement). *At the discretion of the patient or caregiver

Figure 1 shows a schematic overview of the study. The Standard Protocol Items: Recommendations for Interventional Trials (SPIRIT) checklist is provided in Additional file 1. Each patient is involved in the study for 1 year. All patients visit the surgery clinic for an initial visit. Subsequently, patients randomized to the laser group are seen for additional visits for laser treatments every 4–6 weeks until they receive a total of five treatments. Patients in the control group have monthly follow-up at 1, 2, 3, and 4 months by phone or email with the option to schedule additional in-person visits as desired. We continue to follow up with all participants at 6, 9, and 12 months from the initial treatment. Study staff conduct data collection at each follow-up time point. Figure 2 details the schedule of enrollment, interventions, and outcomes assessed at each time point.

#### Post laser treatment follow-up

Immediately following each laser treatment and 24 h after, the patient is asked to rate their pain from 1 to 10 utilizing the numeric rating scale (NRS). The immediate postprocedure pain score is obtained in person at the conclusion of the laser procedure, while the 24 h pain score is communicated to the study team by text message, email, or phone call the following day.

#### Follow-up at 1, 2, 3, and 4 months

The patient and legal guardian complete surveys assessing healthcare-associated disability, pain, pilonidal disease management, and emergency department/urgent care/physician office/hospital visits that occurred between follow-up assessments. In addition, the CATIS is completed at the 2 months follow-up, the parent and child disease-related stigma scales are completed at the 3 months follow-up, and the ageappropriate Child and Parent PedsQL™ is administered at the 4 months follow-up. The healthcare satisfaction questionnaire is administered at the 1 and 4 months follow-ups. These surveys and questionnaires are completed at the in-person visits for patients in the laser group and by either phone or email/web-based survey for patients in the control group (unless they scheduled an in-person visit for any of these follow-ups, in which case they are completed at the in-person visit).

#### Follow-up at 6, 9, and 12 months

Each participant receives an email or telephone call at 6, 9, and 12 months after their initial visit. Follow-up is conducted by email/web-based survey or by phone with a member of the research team from NCH. The patient and legal guardian complete surveys assessing disability days, pain, pilonidal disease management, and emergency department/urgent care/physician office/hospital visits that occurred between follow-up assessments. At 6 and 12 months, the age-appropriate Child and Parent PedsQL™ and the CATIS are administered. At 9 months, healthcare satisfaction and disease-related stigma scales are administered.

#### Medical record review

A medical record review is performed for each patient by a trained member of the research team at the 1 year follow-up, to collect the data detailed in Tables 1 and 2. We record all episodes of care related to pilonidal disease including inpatient and outpatient encounters. If the medical record and patent/caregiver report are discordant, we will ask the patient/caregiver about any discordant episode. If the reports remain discordant, the medical record documentation of medical endpoints (e.g., disease recurrence, wound breakdown) is used in the data analyses.

#### Data collection procedures

All data are collected in a central REDCap database housed at NCH. To minimize variation and improve the consistency of the interpretation of clinical information, we utilize standardized data collection forms with established definitions for all variables. Outcome assessors are blinded to the primary outcome at the 6 months and 1 year time points. Unblinding will only take place in the event of a clinical emergency.

### Statistical considerations

#### Sample size and power

The sample size needed to assess the primary outcome of the proportion of patients with recurrent pilonidal disease at 1 year is based on (1) previous published studies and institutional data on pilonidal disease recurrence rates and (2) the efficacy of laser hair depilation treatment to reduce pilonidal disease recurrence in previous studies and our institutional pilot study. In this RCT, the recurrence rate within 1 year is expected to be a minimum of 12% in the control group and a maximum of 2% in the laser group. Based on these estimated recurrence rates, under a group sequential design with one interim and one final analysis, an overall type I error rate (two-sided) of 5%, and power of 80%, the sample size required for this trial is 122 patients in each treatment group. Assuming a 10% drop-out rate over the course of the 1 year follow-up, we will plan to enroll 136 patients in each group for a total of 272 patients.

#### Planned interim analysis

An interim analysis is planned when one quarter of the planned total patients (34 patients in each group) have completed their 1 year follow-up, and a final analysis will be performed when all patients have completed 1 year of follow-up. These two analyses have been unequally spaced in order to ensure that the interim analysis is performed before study enrollment is complete.

Throughout the trial, the rate of pilonidal disease recurrence at 6 months and 1 year and the tolerance of laser treatments are monitored for safety. Tolerance to the laser treatments is monitored by examining for study-related serious adverse events (SAEs), which include severe pain (defined as a maximum pain score of 8 or above during or within 24 h of treatment) or second degree burns. The rates of SAEs are calculated after every group of 10 patients has completed the course of treatment. The percentage of patients with either a second degree burn or a maximum pain score of 8 or above during or after treatment is estimated. If the lower 95% confidence limit of this proportion exceeds 10%, the trial will be stopped. The 10% level was chosen based on input from patients involved in our feasibility and tolerability pilot in which they expressed that even if they had experienced acute pain or a minor burn, they would likely continue receiving therapy, as these both are self-limited events with minimal long-term morbidity. These rates are regularly reported to the Data and Safety Monitoring Committee (DSMC).

#### Analysis methods

All patient baseline demographics and clinical characteristics will be described and summarized overall and between treatment groups. The balance in these characteristics across treatment groups will be studied and reported. Data on all pre-treatment characteristics (including demographics, race, ethnicity, socioeconomic variables, body mass index (BMI), clinical characteristics, laboratory values, previous episodes of disease, and previous procedures performed) will be collected from the patient, caregiver, and medical record at the time of enrollment. This will allow robust data capture with minimal missing data.

#### Analytic plan

The means/medians and standard deviations/interquartile ranges of all baseline demographic and clinical variables will be evaluated in the total study sample and will be compared between groups using *t* test or Mann-Whitney *U* tests for continuous variables and chi-square tests for categorical variables. For the primary outcome of recurrence within 1 year, we will calculate this proportion and its 95% confidence interval using the Wilson method in both groups and compare this proportion between groups using a Fisher exact test. All outcome comparisons will be conducted using an intention-to-treat (ITT) analysis approach, wherein patients’ data are analyzed according to their randomized treatment assignment. This analysis is hypothesis-driven with an expected decrease in the rate of pilonidal disease recurrence from 12% in the control group to 2% in the laser group.

Heterogeneity of treatment effects will be evaluated for both primary and secondary outcomes across four factors of interest: episodes of previous disease (1, 2, ≥ 3 episodes), previous surgical excision performed (yes/no), gender (male/female), and BMI (under or normal weight vs. overweight or obese). Treatment effect heterogeneity will be explored by evaluating these factors as potential effect modifiers by including each in a model to include the main treatment effect, the main factor effect, and the interaction term for the treatment by factor [25]. Treatment effects will be estimated for each level of the factor and compared across these groups. Identification of effect modification will be made through tests of interaction in these models, which control the family-wise error rate of each of these comparisons at the 1% level (translating to a maximum family-wise error rate of 9%).

Analyses of secondary outcomes will also follow an ITT approach. Categorical secondary outcomes (e.g., complications) will be assessed using estimated proportions and confidence intervals and compared between groups using Fisher exact tests or Pearson chi-square tests as appropriate, similar to the primary outcome. Continuous secondary outcomes (e.g., HRQOL) will be assessed using medians and interquartile ranges and compared between groups using Mann-Whitney *U* tests. These are hypothesis-driven analyses, with the laser group expected to have less disability for both the patient and caregiver, higher HRQOL scores, higher healthcare satisfaction scores, more favorable disease-related attitudes and perceived stigma, lower rates of pilonidal disease-related complications, lower rates of incision and drainage, lower rates of surgical excision, lower rates of postoperative complications, and higher compliance with treatment. We will give specific consideration to the potential for varying treatment effects across groups defined by the four patient characteristics described in the preceding paragraph. In further exploratory analyses, we will examine the complete longitudinal trajectory of both patient- and guardian-reported HRQOL, satisfaction with healthcare, and disease-related attitudes and perceived stigma through the use of linear mixed effects models [26]. In addition, both primary and secondary endpoints will be examined, though not formally statistically compared, across various subgroups defined by patient socioeconomic status (SES) and demographic characteristics, including race, ethnicity, insurance status, household annual income, and number of household residents. These subgroup analyses will be exploratory rather than confirmatory.

### Safety monitoring

The attending surgeons and staff in the surgical clinic are managing the clinical care of the participants. Vital signs, physical exam, and pain scores are assessed per nursing protocol and surgical service standards. An attending surgeon assesses each patient before and after each treatment. A research team call schedule is maintained, with a member of the study team available by pager 24 h per day. Any suspected adverse event (AE) identified by this person is discussed with the study principal investigator (PI). A DSMC has been formed and will meet every 6 months throughout the period during which patients are being recruited and through their first year of follow-up. The DSMC will review data provided by the primary study statistician and other study staff involved in data management and analysis. The study PI will be made aware of all AEs as they occur, and a quarterly review of all AEs that occur in the trial will be performed by the study team. All unexpected non-serious AEs and SAEs relating to participation in the study will be reported verbally and in writing to the study PI and the NCH Institutional Review Board (IRB). The verbal report will occur within 48 h of the occurrence. The written report of a SAE (e.g., death or a life-threatening AE) will be reported within 7 days.

### Study retention

Study retention is promoted by providing Mastercard® ClinCard incentives for completing follow-up assessments, by using regularly scheduled short interval contact with the participants throughout the 1-year duration of the study, and by providing multiple methods for follow-up including phone, email, and text.

## Discussion

The results of this study will be important, because the incidence of pilonidal disease in the adolescent population is high, and few effective treatments exist presently. Recurrence rates after less invasive measures, including antibiotics and/or incision and drainage, have been reported to be as high as 30% [27]. Recurrence rates after surgical excision, considered to be the gold standard treatment for recurrent pilonidal disease, are also high [27, 28]. In addition, patient morbidity after surgical excision is considerable, with a significant percentage of patients suffering from severe wound complications postoperatively [29]. Thus, treatments are clearly needed to prevent the significant morbidity caused by not only the disease, but also the surgical treatments currently available. The success of laser hair depilation to reduce pilonidal disease recurrence may prevent many patients from developing chronic infections and wounds, thereby reducing the number of patients subjected to the significant morbidity of this disease. Positive results from this study may transform the treatment paradigm of pilonidal disease—from one in which patients, families, and physicians anticipate recurrences, to one in which a less invasive office-based therapy may eradicate the disease entirely.

Multiple groups have acknowledged the efficacy of laser hair depilation to prevent recurrence of pilonidal disease [11, 19,18, 30–32, 33]. However, these groups believe that additional well-controlled prospective studies are critical for establishing its effectiveness prior to recommending it to all patients. This study aims to determine the effectiveness of laser hair depilation to decrease the recurrence of pilonidal disease in adolescents and young adults. To maximize the generalizability of our results, all patients with mild to severe disease are being enrolled. Results from this study should be able to be widely disseminated and applied to the treatment of pilonidal disease in adolescent and adult patients.

### Trial status

This RCT began enrolling patients in September of 2017. Active enrollment is open and over 50 patients have been enrolled as of 10/25/2018.

## Additional file

Additional file 1: SPIRIT 2013 checklist: recommended items to address in a clinical trial protocol and related documents. (DOC 121 kb)

## Abbreviations

BMI: Body mass index
CATIS: Child Attitude Toward Illness Scale
DSMC: Data and Safety Monitoring Committee
HRQOL: Health-Related Quality of Life
ITT: Intention to treat
NCH: Nationwide Children’s Hospital
NRS: Numeric rating scale
OSU: Ohio State University
PedsQL: Pediatric Quality of Life
SAE: Serious adverse event
